# Supplementary material for: A Global Screen for Assembly State Changes of the Mitotic Proteome by SEC-SWATH-MS
Source: Cell Syst. 2020 Feb 26;10(2):133–155.e6. doi: 10.1016/j.cels.2020.01.001 (PMC7042714; doi:10.1016/j.cels.2020.01.001)

Q9P0U4 | CXXC1_HUMAN | CXXC1 CFP1 CGBP PCCX1 PHF18
Monomer MW [kDa]: 75.712 Monomer expected elution fraction: 43

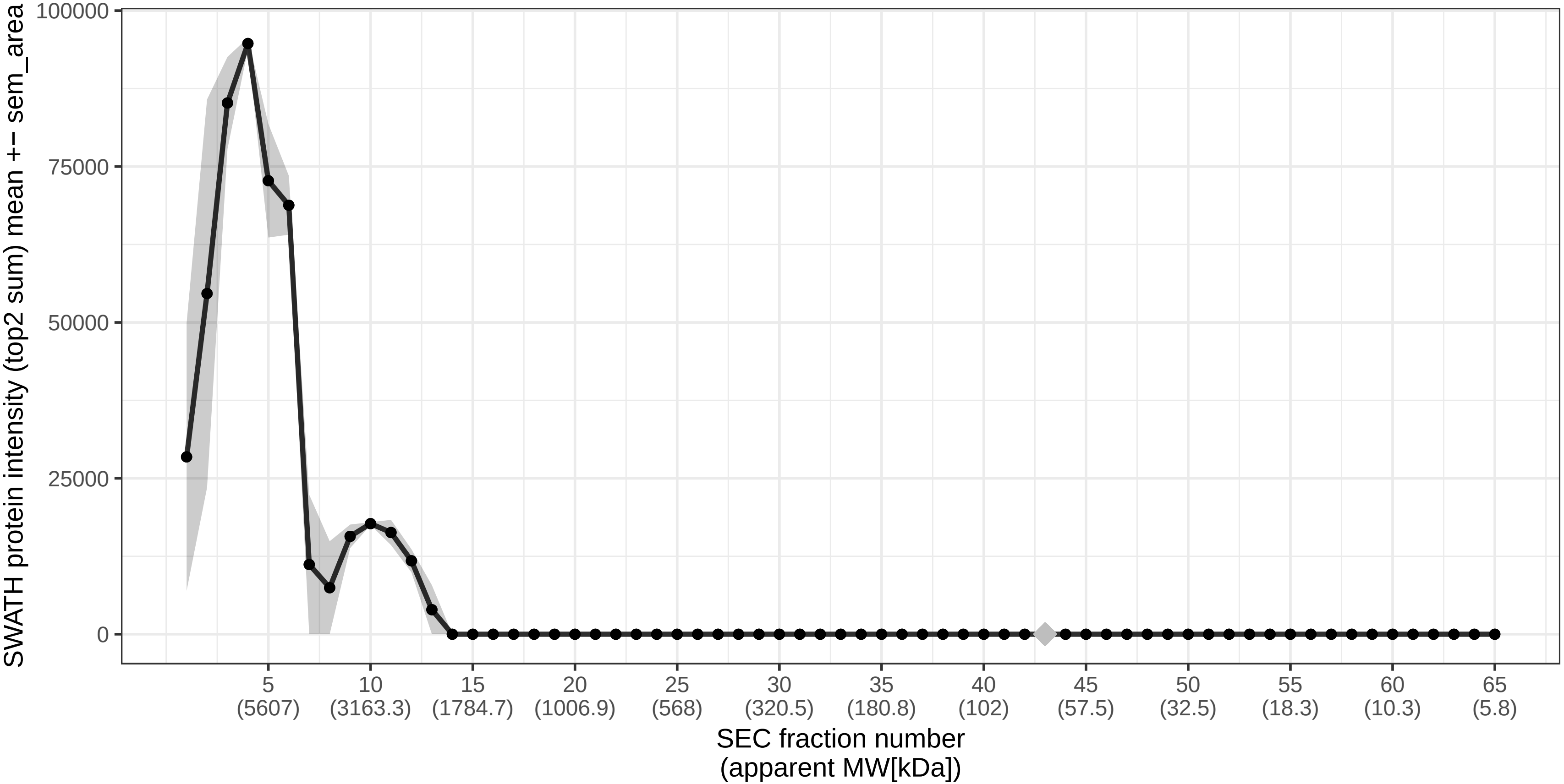

Supplement: Data S1. SEC-SWATH-MS Protein Chromatograms, Related to Figure 1 [file mmc6.zip › SECchrom_Q9P0U4_CXXC1_HUMAN_CXXC1_CFP1_CGBP_PCCX1_PHF18.pdf]
